# Nurses' Experiences of the Prerequisites for Implementing Family-Centered Care to Prevent Pediatric Delirium

**DOI:** 10.1097/NUR.0000000000000842

**Published:** 2024-08-05

**Authors:** Tiina Saarenpää, Miia Jansson, Heli Kerimaa, Riitta Alanko, Outi Peltoniemi, Miikka Tervonen, Tiina Lahtela, Tarja Pölkki

**Affiliations:** **Author Affiliations:** Professor (Dr Pölkki), Research Unit of Health Sciences and Technology, University of Oulu, Finland (Saarenpää, Drs Jansson and Kerimaa, and Alanko); Royal Melbourne Institute of Technology (RMIT University), Australia (Dr Jansson); and MRC Oulu, Oulu University Hospital and University of Oulu (Drs Jansson, Kerimaa, Peltoniemi, Tervonen, and Pölkki); Department of Children and Adolescents, Oulu University Hospital (Drs Peltoniemi and Tervonen, and Lahtela); and Research Unit of Clinical Medicine, University of Oulu, Finland (Drs Peltoniemi and Tervonen).

**Keywords:** family-centered care, nursing, pediatric delirium, pediatric intensive care, qualitative research

## Abstract

**Purpose:**

The aim of this study was to describe nurses' experiences of the prerequisites for implementing family-centered care to prevent pediatric delirium.

**Design:**

The research employed a qualitative, descriptive study design.

**Methods:**

A total of 10 nurses working in the pediatric intensive care unit at 1 university hospital participated in the study. The quality data were collected using individual semistructured interviews, and the data were then analyzed by inductive content analysis.

**Results:**

The prerequisites for implementing family-centered care to prevent delirium among pediatric patients consisted of 30 subcategories that were grouped into 11 generic categories. The generic categories were further grouped into 5 main categories: (1) an environment that supports family presence, (2) psychosocial support for the family, (3) individual family involvement, (4) family participation in shared decision-making, and (5) nurses' professional competence.

**Conclusions:**

According to the nurses' experiences, the implementation of a family-centered approach to preventing delirium in pediatric patients requires creating a supportive environment for families, providing psychosocial support, encouraging family involvement in decision-making, and ensuring that all nurses have the necessary skills.

Pediatric delirium (PD) is a frequently overlooked issue that is rather common among children in intensive care.^1–6^ It is a form of brain dysfunction or altered mental status that causes significant dysfunction and anxiety.^7–9^ Acute PD can be hypoactive (lethargic behavior, lack of attention), hyperactive (increased psychomotor activity), or a combination of these two.^2,6^ The prevalence of PD has been found to be highest during the first 2 days of intensive care,^6^ as this period is characterized by disruptions to the child's normal sleep-wake rhythm and other familiar routines.^2,7^ According to other reports, PD is associated with certain treatments, such as mechanical ventilation.^2,7^ Patients who experience delirium, which can involve severe confusion, suffer from stress,^10^ which also affects family members, nurses, and the multidisciplinary care team.^1^ Moreover, another symptom of delirium can be frightening hallucinations or delusions, which involve the risk of pediatric patients attempting to harm themselves.^1^

The early identification and prevention of PD syndrome improves outcomes,^6,7^ lowers healthcare costs,^6–8^ and reduces mortality rates.^7,8^ Hospitals can address the risk factors for delirium syndrome by identifying predisposing factors and changing practices,^6,11^ with such initiatives producing promising results.^4,5,12^ Children who require intensive care have many risk factors for delirium, including age (children younger than 2 years old), comorbid medical conditions, cognitive impairment, excessive sedation, and psychoactive medications.^3,7^ Physical disability and immobility,^2,3,7^ along with infectious^3^ and neurological diseases,^7^ can also increase the risk of experiencing delirium. The latest research has shown that hospitals should reduce sleep disturbances, excessive pain management approaches, and benzodiazepine use^2,7,10,13^ to minimize the prevalence of delirium among pediatric intensive care unit patients.

Family-centered care emphasizes the planning, implementation, and evaluation of care in collaboration with members of a patient's family^14,15^ and has been shown to increase the well-being of patients and their families.^15^ This approach is pivotal to fostering support for families in the pediatric intensive care environment.^16^ An important part of family-centered care is communication; as such, nurses should proactively consider which information about a child's health fulfills the needs of family members.^17,18^ This, however, should not be one-way communication, and parents can contribute to improving communication.^11,19^ Moreover successful family-centered care requires respect, collaboration,^20,21^ and actively aligning the care culture with a family-centered approach of care.^20^

Previous studies have focused on experiences from both adult and pediatric intensive care units, along with the parenteral perspective, to explore how hospitals can reduce the emotional distress associated with intensive care to effectively decrease the incidence and duration of delirium.^4,15,22–24^ Nevertheless, healthcare professionals need to first understand the concept of family-centered care in the context of pediatric intensive care if we hope to witness improvements in patient and family satisfaction and care outcomes.^15,20^ Thus, more research is needed on what is required for the implementation of family-centered care in the pediatric intensive care setting.^15^ The aim of this study was to describe nurses' experiences of the prerequisites for implementing family-centered care to prevent PD. The insights shared in this article can be used to develop education for healthcare staff and e-services for the prevention of delirium syndrome.

## METHODS

### Study Design

A qualitative descriptive study design was chosen to gain a deeper understanding of nurses' experiences with the implementation of family-centered care to decrease PD.^25–27^

### Research Environment and Participants

The study involved interviews with 10 nurses working in the pediatric intensive care unit at 1 Finnish university hospital. The pediatric intensive care unit provides care for newborn and young people between the ages of 0 and 16 years, for example, intensive care or monitoring due to respiratory distress, metabolic disease, or infections. The unit also treats children who have experienced trauma, poisoning, burn, and neurological or surgical conditions who need monitoring or pain management. The nurses who participated in the study were between 23 and 61 years old and were selected based on their knowledge of, and experiences with, the research topic. The inclusion criteria were as follows: (1) working in one of the pediatric intensive care units where children may suffer from delirium and (2) willingness to participate in the study. Summer workers or occasional single-shift workers were excluded from the study. The interviewees were recruited with the help of a person working in the same unit and approached by email.

### Data Collection

Semistructured interviews were conducted from May to October 2022 to collect data. The main interest was the implementation of family-centered care to prevent PD during intensive care, the prerequisites for implementation, taking patient needs into account, disease and treatment, and environmental factors.^28–32^ The themes of the interviews were based on Smith's^20^ (2018) conceptual analysis of family-centered care, and related to the prerequisites and characteristics of family-centered care for the prevention of PD. The interviewer asked participants further questions if they felt as though this was necessary to clarify matters. These questions were about the characteristics of family-centered care in terms of involvement, support, respect, and communication to prevent PD. The interview structure was slightly modified based on their feedback. The interviews were conducted remotely and recorded. To ensure privacy, the interviews were conducted in a quiet private space, as the time spent in the interviews was considered working time. Both the interviewer and the participant turned on their video cameras at the beginning of the interview so that they could become familiar with one another. The interviewer and participant later turned off the video function and generally kept it switched off during the interviews. The interviews lasted between 29 and 49 minutes. Interview data were collected until data saturation was reached; this was noticeable after the ninth interview.

### Data Analysis

The data were analyzed using inductive content analysis. This meant that although the themes of the interviews guided the data collection, they were not used to analyze the data. The interview material was transcribed word-by-word and included only verbal expressions. First, the researcher TS read through the material several times to get an overall picture of the data. The unit of analysis was a set of meanings^33^ related to experiences of family-centered care. The analysis did not consider facial expressions, gestures, voice inflection, or pauses. A preliminary analysis of this material identified a total of 940 reduced expressions, which was indicative of rich data for analysis. Following this, the reduced expressions were grouped into subcategories based on their similarities in content. This process, termed abstraction, continued as long as it was possible to combine the reduced expressions into categories. At the end of this process, the prerequisites for implementing family-centered care to prevent delirium in pediatric intensive care patients consisted of 30 subcategories. Subcategories were further grouped and named as descriptive in content into 11 generic categories, and these in turn were grouped into 5 main categories. One researcher (XX) had primary responsibility for data analysis, but the other members of the research team resolved disagreements when they arose. The example of the analysis process that produced the main category “individual family involvement” is shown in Table 1.

**Table 1:** Example of the Inductive Content Analysis Process, Which Yielded the Main Category “Individual Family Involvement”

Examples of Quotations	Reduced Expression	Subcategory	Generic Category	Main Category
“...at least here we actively involve parents in the treatment...and of course it depends on the age of the child....” (101)	Parental involvement in care, according to the age of the child	Parental involvement in care	Enabling participation	Individual family involvement
“Let's ask the parents to come to the so-called morning treatments, for example, to wash....” (103)	Asking parents to participate in the washing			
“...depends a lot on the care of the child...especially children with developmental problems....” (105)	Participation in care is possible regardless of treatment status			
“...most parents want to participate, of course they want to participate in their child's care....” (106)	Taking into account the parents' wishes in terms of participation in care			

### Ethical Considerations

The research followed good scientific practice on the ethical principles, both of which are required in research involving human subjects.^34^ A local ethics committee also approved the study (EETTMK:86/2021). After receiving clear information about the purpose of the interview, along with data processing, recording, and storage, each participant provided written informed consent. Personal data were collected only for the interview. Data collection followed the guidelines of the Data Protection Act (1050/2018) and EU General Data Protection Regulation (EU 679/2016).^35,36^ The interview data were processed pseudo-anonymously, and interviewees are referred to by their randomized ID in the article. All of the recordings were destroyed after transcription.

## RESULTS

The prerequisites for implementing family-centered care to prevent PD consisted of 5 main categories, namely, (1) an environment that supports family presence, (2) psychosocial support for the family, (3) individual family involvement, (4) family participation in shared decision-making, and (5) nurses' professional competence (Table 2).

**Table 2:** Inductive Content Analysis Results Concerning Nurses' Experiences of the Prerequisites for Implementing Family-Centered Care to Prevent Delirium Among Pediatric Patients

Subcategory (n = 30)	Generic Category (n = 11)	Main Category (n = 5)
Visiting hours Visitors	Flexible visiting practices	An environment that supports the presence of the family
Physical facilities Privacy of the premises	A favorable environment	
Emotional support for the family Allowing family members time to rest	Emotional support	Psychosocial support for the family
Support from specialist workers Support from social services	Social support	
Parental involvement in care Parental involvement in hospital examinations Parental involvement in the report	Enabling participation	Individual family involvement
Parental support for their child Respect for uniqueness	Respect for individuality	
Decision-making Cooperation skills Respecting parents' wishes Making use of parents' expertise	Cooperation in decision-making	Family participation in shared decision-making
Keeping in touch Communication Listening Open atmosphere	Two-way communication	
Collecting feedback Getting feedback Making use of feedback	Continuous development of family-centered care	
Continuing education Induction	Developing competences	Nurses' professional competence
Parental encouragement Parental guidance Parental involvement	Guidance skills	

“An environment that supports family presence” comprised 2 generic categories: flexible visiting practices and a favorable environment. Unlimited visiting hours made it possible for parents to stay with their child as long as they wished, as described in the following quotation:

Family presence is enabled by the fact that there are no kind of visiting hours in the unit...but there are also situations where the parents do not want to go home at night. (102)

However, the participating nurses did highlight some instances in which the number of visitors had to be limited to acute situations or seasons in which viral infections are more common. A favorable environment includes good physical facilities, as shared in the following quotation:

I think that's probably going to change when we move to the new facilities, the new hospital, where there are single rooms for the child and the family. (108)

Privacy in the facilities was enhanced by the use of partition curtains between patient beds and attention to privacy through the use of headphones.

“Psychosocial support for the family” included 2 generic categories: emotional support and social support. Based on the nurses' experiences, emotional support included an emotional response to family members during frightening situations, as outlined in the following quotation:

The ventilator patient, it scares a lot of people because there are so many tubes and there are intubation tubes and everything.... (110)

Allowing family members time to rest mostly manifested as providing adequate sleep and breaks. When discussing social support, nurses described that support and guidance from either a specialist worker or social worker, who are experienced at helping parents spend time with their child in the unit, as described in the following quotation:

If a newborn child has to go into intensive care, the father should at least not waste paternity leave days, he can get either medical leave or a D-certificate...time off for the period of the child's stay at the hospital. (101)

“Individual family involvement” included 2 generic categories: enabling participation and respect for individuality. The interviewed nurses described that enabling participation comprises parental involvement in care, parental involvement in examinations, and parental involvement in the report of their child's hospitalization. Parental involvement was seen as an opportunity to reassure the child, as shown in the following quotation:

They can participate as much as they can...some participate more, some less, some have to be pushed to remember to let their children rest and relax.... (107)

When asked to elaborate on parental involvement in the report, the participants cited opportunities to attend a doctor's round and getting acquainted with nurses' rotation reports. Respect for individuality was divided into parental support for the child and respect for uniqueness. Nurses reported that parental support for the child often took the form of reassurance, comfort, and security, and attention should be paid to the uniqueness of each child, as outlined in the following quotation:

In everyday activities and in shaping the child's everyday life, we always try to be like at home as much as possible...the routine of the day would be the same. (105)

“Family participation in shared decision-making” comprised 2 generic categories: cooperation in decision-making and 2-way communication. Collaboration in decision-making included decision-making, cooperation, listening to the wishes of parents, and making use of parents' expertise. This can be noted in the following quotation:

The possibility of cooperation when making treatment restrictions. (103)

Nurses described how the possibility for cooperation was based on the willingness and ability of parents to cooperate. Respecting parents' wishes was seen as an opportunity to actively listen to the parents' desires. In addition, the use of parental expertise was evident in relation to the child's expressions, pain, developmental stage, and culture. Two-way communication involved discussions, communication, information flow, listening, and an open atmosphere with the families. The participating nurses highlighted how communication was critical to setting up video calls with family members and calling upon translation services when they were deemed necessary. This is described in the following quotation from a participating nurse:

...take into account the flow of information between caregivers about the patient's issues, habits and routines in the report. (106)

An open environment was seen as a space or setting that inspires feelings of trust, security, and tolerance.

“Nurses' professional competence” consisted of 3 generic categories: continuous development of family-centered nursing, competence development, and guidance skills. The nurses discussed how they take into account various feedback when developing family-centered care, with one of the participants sharing:

...take into account the issues to be developed in relation to promoting inclusivity in care. (107)

Competence development included in-service training and induction, with one of the interviewed nurses specifying:

Education...how to face people and families in different life situations...which would increase the capacity, and also perhaps the courage, for nurses in certain situations to face challenging relatives.... (104)

In addition, the interviewed nurses highlighted how guidance skills are an important part of their professional competence. This competence area includes encouraging parents, guiding parents, and involving parents in their child's care, with one of the interviewees stating:

It's a bit like being urged to go on breaks sometimes, because sometimes it seems that the parents are here from morning till night without eating and drinking, and without even going to the toilet...so we remind them to go rest and eat.... (109)

## DISCUSSION

This study found that the successful implementation of a family-centered care for preventing delirium in pediatric patients requires the creation of a supportive environment for families, providing psychosocial support, encouraging family involvement and participation in decision-making, and ensuring that nurses possess the necessary professional competencies.

The interviews with nurses highlighted the importance of an environment that supports family presence, along with flexible visiting practices that allow for family presence.^37^ Family presence contributes to patient well-being by reducing the traumatic experience of illness and enables participation in treatment.^37^ Previous reports of limiting visits by family members during a pandemic^38^ or in emergency situations^13^ agree with what was stated in the interviews of this study; that is, there are certain cases in which access to pediatric patients must be limited due to health and safety reasons. The results also showed that a favorable environment takes into account the needs of the family, including physical presence^13,20^ and cultural values.^13^ According to the interviews, a hospital that is currently under development will allow for a bed to be placed next to the pediatric patient in single rooms. In line with previous studies, the participating nurses shared how it is important for parents to be near critically ill child^13,39^ and that nurses should pay attention to parents' individual needs when discussing sensitive issues at the intensive care setting.^39^ Previous studies have reported how investments into noise reduction^18,39^ can improve the hospital environment by supporting normal sleep and wake patterns and adherence to familiar routines, for example, sounds, lights, and comforts at different times in the day.^2,7,18^ Notably, the research presented by Bosch-Alcaraz et al (2020) and Petersson et al (2019) explicitly state that intensive care settings have a far greater need for such developments than other hospital settings. The results showed that nurses' experiences of what it takes to implement family-centered care in pediatric patients emphasized an environment that supports the presence of the family to prevent PD.

The results emphasized how a nurse should provide initial emotional support to parents coping with their child being admitted to intensive care. The results showed that family support is a prerequisite for implementing family-centered care in the prevention of PD. This finding seems logical, as nurses spend most of their time with the child and their family. However, it should be noted that nurses are not always able to identify the support needs of family members.^15^ According to Bettencourt and Mullen (2017), the importance of providing psychosocial support to a child and family affected by delirium cannot be understated. Previous research has found that parents who trust the nurses in charge of care find it easier to take a break^21,39^ and that this parent-nurse trust is strengthened by periodic updates on the child's condition.^21^ Moreover, the fear experienced by parents can be reduced by access to information concerning the medical equipment, monitors, instruments, and medicines.^18^ The nurses interviewed in this study identified a barrier to receiving adequate emotional support in the ward setting. This may be explained by the recent implementation of discussion support at the study hospital, which was mandated due to identified shortcomings. Previous research has already touched upon the importance of multidisciplinary well-being services for parents of pediatric patients in intensive care.^16^ However, it should be taken into account that this is a delicate situation, as Alzawad et al (2022) have explained how parents may feel inadequate based on more time spent with 1 child rather than the siblings.

The involvement and individuality of the patient's family was also emphasized in the results of this study as a prerequisite for the implementation of family-centered care for the prevention of PD. For instance, the interviewed nurses noted how it is important to involve family members in the small tasks of care,^13,18,36^ such as diaper changes,^13,18,39^ along with bathing,^13^ feeding,^18^ and supporting the child.^36^ Parents have previously stated how they are grateful when nurses involve them in care.^18^ The analysis of the interviews confirmed that parents have an important role, and this was especially relevant for younger patients and children with developmental delays. This can be explained by parents being better at interpreting their alienated or developmentally delayed child, which facilitates better communication. Family presence has also been reported to provide comfort and reassurance to a critically ill child.^36^ The results also highlighted that each family has unique habits, customs, and routines; as such, nurses who have the opportunity to get to know a family's background can be expected to provide better care for the child.^39^ Nevertheless, it should be noted that several interviewees stated that some family members may be afraid to get involved, potentially due to a fear of being incompetent in medical care.

Our results highlight the importance of family involvement in shared decision-making as a prerequisite for implementing family-centered care for the prevention of PD. Nurses have a unique role in ensuring that the families of pediatric patients receive accurate, understandable information^13^ and are kept up-to-date on the condition of their child.^40^ According to the results presented in the articles by Hill et al (2018), it is important for a doctor to explain things in a way that the family can understand. The results of the present study suggest that parents in difficult situations should receive ample opportunities for communication and discussion. In addition to nurses, previous research has stated that the doctor who is in rotation should also proactively provide parents with relevant information.^15^ Challenges surrounding the information flow between healthcare professionals and parents could be caused by the parents' absence from medical visits, lack of a common language, or lack of cooperation. According to the study of Smith et al (2018), mutual respect, cooperation, and open communication are all key to the success of family-centered care. Moreover, listening to parents can ensure that families receive enough information.^41^ The analysis of the collected interviews also suggests that parents should continue to make their wishes known and use their expertise to help their children. However, some of the participating nurses shared that challenges in meeting parents' wishes and using their expertise may occur when parents withdraw because of the difficult situation.

The results of our study demonstrate that nurses must be adept at continuous follow-up and review of patient-centered care of the prerequisite for implementing family-centered care to prevent PD. More specifically, feedback should be seen as a resource and used to identify both good practices and areas for improvement. The implementation of family-centered care is influenced by health policy decisions and programmers,^14,39^ which have recently shifted healthcare culture toward a more family-centered model of care.^20,39^ The results of this study highlighted the importance of encouraging, guiding, and involving parents in their child's care. In some cases, care can progress faster when the family is not involved. On the other hand, the results of this study emphasize how family involvement results in good experiences and strengthens professionals' family-centered care skills. Nevertheless, it should be stated that nurses are primarily responsible for the identification and management of PD syndrome^1^ to prevent and reduce the duration of this complication^4,42^ in critically ill children.^1,14,42^ As such, the presented results stress that every nurse who works in the intensive care setting needs to be skilled at delivering family-centered care; these skills can be strengthened through further education. In addition, organizations should ensure that all of the nurses working with critically ill children understand how to notice delirium^39^ and alert family members, and use multidisciplinary approaches to treat delirium syndrome.^1,16,40,43^

### Trustworthiness

The trustworthiness of the research was assessed according to the guidelines of Lincoln and Guba^44^ (1985). The interview framework used in the study was tested before data collection, and the interviewees were selected based on their knowledge of the research topic.^45^ *Credibility* was assessed based on data saturation. The collected data were considered sufficient for investigating the study topic, as data saturation was achieved after the ninth interview. In addition, 2 researchers with backgrounds in intensive care and patient monitoring carried out the thematic interviews; this strengthened the objectivity of the research, as 1 researcher's preconceptions could not overly influence the analysis. Credibility could have been improved if the interviews could have been conducted face-to-face, even though the atmosphere of the virtual interviews was open and confidential. It was decided to collect the data remotely as the interviewers came from different cities than the participants. *Dependability* was assessed as a whole during data collection, with an overarching aim of having the concepts and interpretations completely reflect the views of the interviewees. The chosen unit of analysis was the set of meanings with experiences of family-centered care. To improve dependability, the results of the content analysis could have been presented to the participants for confirmation, but the data analysis results stayed within the research team to maintain confidentiality. Three members of our research team critically evaluated the analysis process, checking that the interview data were correctly coded and that the categories were based on the data. To help the reader follow the analytical process, 2 tables describing the steps of the data analysis were included in the report. *Confirmability* was assured by providing a detailed description of the research process, along with a discussion of how the results compared to the findings presented in relevant references. *Authenticity* was verified by providing actual, verbatim quotations from participants in the text. The *transferability* of the results can be assessed by considering their relevance in another context^45^ with similar patients and treatment practices. The COnsolidated criteria for REporting Qualitative research-32 checklist was used to report the findings of this qualitative research,^46^ with the overall goal of increasing transparency.

## CONCLUSIONS

The presented findings highlight that the implementation of a family-centered approach to preventing delirium in pediatric patients requires creating an environment that supports the presence of the family, allowing the child to be present, and encouraging individual participation alongside the child in treatment, examinations, and decision-making. The inclusion of the entire family supports the child in their treatment journey and is pivotal to respecting their uniqueness. Providing parents with psychosocial support strengthens their ability to cope with the current situation, as well as manage other practical family matters. This support provides parents with resources and can foster a favorable environment in which the parents and child can interact. Developing nurses' competence in family-centered care will help strengthen their ability to prevent and effectively manage delirium syndrome in children.
